# Revisiting the forgotten remnant: Imaging spectrum of Meckel’s diverticulum

**DOI:** 10.4102/sajr.v26i1.2431

**Published:** 2022-07-19

**Authors:** Manish Kumar, Priya Singh, Priti Kumari, Rohit Kaushik

**Affiliations:** 1 Department of Radiodiagnosis, Mayo Institute of Medical Science, Barabanki, India; 2 Department of Radiodiagnosis, BPS Government Medical College for Women, Sonepat, India; 3 Department of Radiodiagnosis, House of Diagnostics, New Delhi, India

**Keywords:** Meckel’s diverticulum, computed tomography, imaging, complications, Meckel’s acute abdomen

## Abstract

Meckel’s diverticulum is a true diverticulum of the alimentary tract occurring resulting from the persistence of remnants of the vitello-intestinal duct. They are often asymptomatic and incidentally diagnosed during surgery. Complications such as intestinal obstruction, diverticulitis, intestinal haemorrhage and perforation may occur with Meckel’s diverticulum, which renders them symptomatic. The clinical and imaging diagnosis of Meckel’s diverticulum is very challenging. As a result of the rare occurrence of complicated Meckel’s diverticulum and the difficult preoperative diagnosis, knowledge of its imaging features is limited. The presented case series describes a spectrum of complications caused by Meckel’s diverticulum and its CT imaging features. It highlights the importance of a high clinical suspicion by carefully searching for a Meckel’s diverticulum on CT in its characteristic location to avoid missing it preoperatively.

## Introduction

Meckel’s diverticulum (MD) is the commonest structural congenital anomaly of the gastrointestinal tract. It is part of the spectrum of abnormalities that occur because of the persistence of remnants of the vitellointestinal duct. It is a true diverticulum of the alimentary tract consisting of all layers of the intestinal wall and lined by normal intestinal mucosa, which frequently contains heterotopic gastric or pancreatic mucosa.^1^ The incidence of MD is about 2% – 3% in the population with a similar occurrence in both sexes; however, it often becomes symptomatic in males. Although it is mainly asymptomatic, a myriad of complications may develop with a lifetime risk of about 4.2% – 6.4%.^2^ The common complications include intestinal obstruction, diverticulitis, intestinal haemorrhage and perforation.

Thus, knowledge of the embryology, anatomy, clinical presentation and diverse complications is of paramount importance. The diagnosis of MD and its related complications is often challenging to establish preoperatively. However, identification of MD and its various complications can be reliably achieved with improved CT scan techniques.

## Case presentations

### Case 1

**FIGURE 1:**
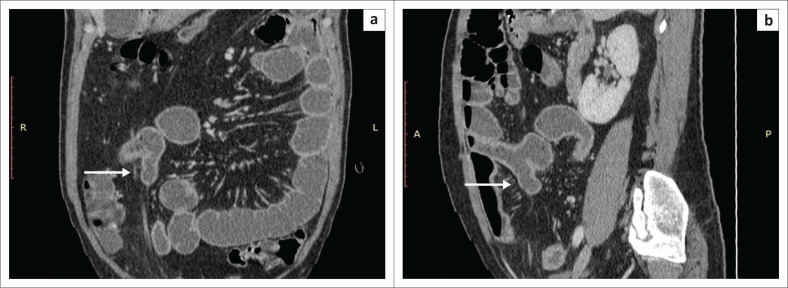
Contrast enhanced computed tomography in the coronal (a) and sagittal (b) planes demonstrating a tubular, blind-ending structure arising from antimesenteric border of the distal ileum with enhancing walls, suggestive of Meckel’s diverticulum (white arrows). No abnormal wall thickening or surrounding fat inflammation was present.

A 25-year-old man presented with complaints of left lower abdominal pain radiating to the back. On ultrasound, multiple renal calculi were present in the left kidney with mild hydronephrosis. A contrast enhanced CT (CECT) and urography were performed for further evaluation, confirming left lower ureteric and left renal calculi with mild left hydronephrosis. In addition, a tubular diverticulum was seen arising from the antimesenteric border of the distal ileum, just proximal to the ileocecal junction (Figure 1). A diagnosis of incidentally diagnosed MD was made. The patient was treated for ureteric calculi and as the MD was asymptomatic, no surgical treatment was performed and follow-up was advised.

### Case 2

**FIGURE 2:**
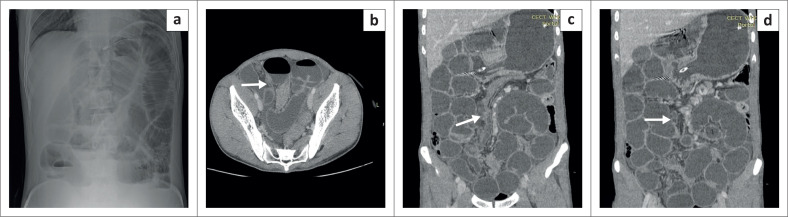
X-ray abdomen (a) erect anteroposterior view shows dilated gas-filled bowel loops with multiple air-fluid levels. Contrast enhanced computed tomography in the axial (b) and coronal (c, d) planes shows dilated small bowel loops with an inflamed Meckel’s diverticulum (white arrows) arising at the level of the transition point.

**FIGURE 3:**
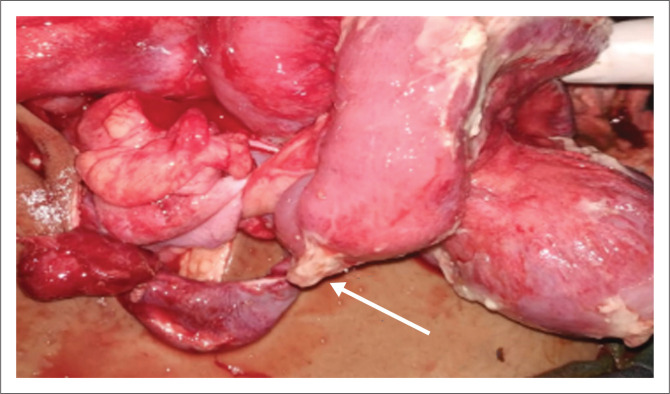
Intraoperative image demonstrated the inflamed Meckel’s diverticulum (white arrow) causing a stricture of the distal ileum resulting in intestinal obstruction.

A 30-year-old man presented with abdominal distension, vomiting and obstipation for three days. On ultrasound, the small bowel loops were dilated and fluid-filled, with to and fro movements. An erect abdominal X-ray demonstrated dilated central bowel loops with multiple air-fluid levels suggestive of small bowel obstruction. The CECT abdomen revealed small bowel dilatation with multiple fluid levels and a transition point at the level of the distal ileum. A tubular blind-ended structure arising from the antimesenteric border was seen at the transition point (Figure 2). The diagnosis of a MD causing small bowel obstruction was made on imaging. The patient immediately proceeded to laparotomy, where an inflamed MD was found, causing a stricture in the adjoining distal ileum (Figure 3). Surgical resection of the MD and small bowel was performed.

### Case 3

**FIGURE 4:**
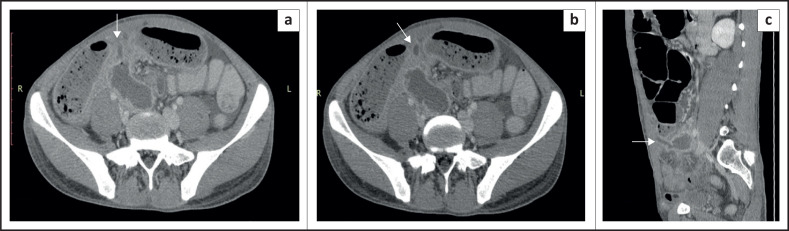
Contrast enhanced computed tomography in the axial (a, b) and sagittal (c) planes revealed a Meckel’s diverticulum (white arrows) with wall thickening and enhancement, surrounded by fat inflammation suggestive of Meckel’s diverticulitis.

A 36-year-old male presented with right lower quadrant and periumbilical abdominal pain for 1 month. Ultrasound of abdomen was within normal limits. Contrast enhanced CT abdomen revealed the presence of a MD with thickened, enhancing walls associated with inflammatory changes in the adjacent fat (Figure 4). The normal appendix was identified separately on CT, resulting in a diagnosis of Meckel’s diverticulitis. The patient underwent laparoscopically assisted trans-umbilical Meckel’s diverticulectomy. Histopathology confirmed the imaging diagnosis.

### Case 4

**FIGURE 5:**
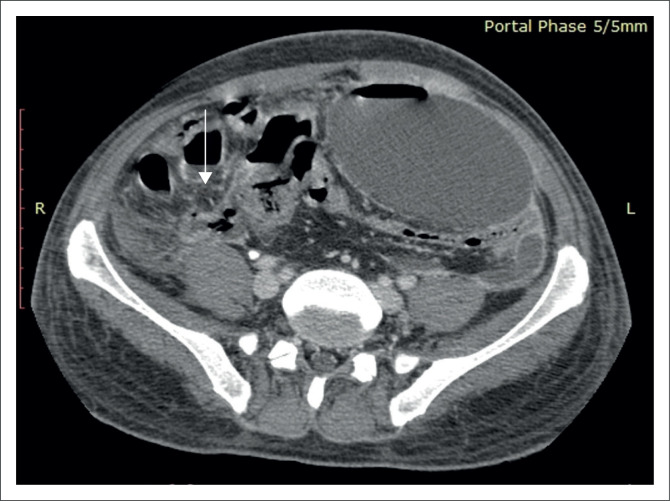
Contrast enhanced computed tomography in the axial plane shows a tubular blind-ending structure arising from antimesenteric border of the distal ileum with surrounding free air suggestive of a perforated Meckel’s diverticulum (white arrow).

A 40-year-old male patient presented with complaints of acute severe abdominal pain and swelling predominately towards the right side, associated with fever and obstipation. His general examination revealed marked tenderness of the abdomen, guarding and rigidity, raising the suspicion of perforation with peritonitis. An erect plain X-ray abdomen revealed free air under the diaphragm. An urgent CECT abdomen was performed, which indicated an inflamed perforated MD arising from the distal ileum with free extraluminal air (Figure 5). Surrounding marked inflammation and fat stranding was seen. An urgent laparotomy was performed, which confirmed the imaging findings.

### Case 5

**FIGURE 6:**
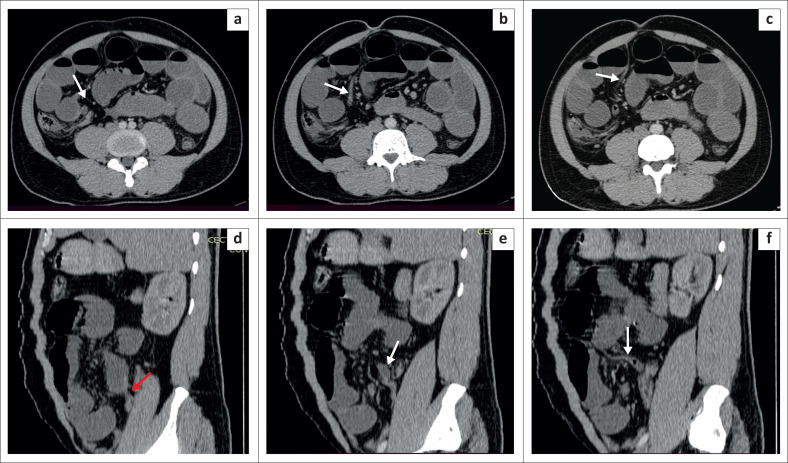
Contrast enhanced computed tomography in the axial (a, b, c) and coronal (d, e and f) planes show dilated small bowel loops with air-fluid levels. An inflamed Meckel’s diverticulum (white arrows) was seen arising at the level of the transition point (red arrow). An enhancing linear band was seen arising from the Meckel’s diverticulum, extending towards umbilicus (white arrow in f).

A 29-year-old male patient presented with acute abdominal pain in the mid-abdomen associated with vomiting. Abdominal X-ray revealed air-filled distended bowel with multiple air-fluid levels in the central abdomen. Contrast enhanced CT abdomen demonstrated dilated small bowel loops with a transition at the level of the distal ileum. A MD was seen at the level of the transition point, with its tip reaching up to the umbilicus (Figure 6). Diagnostic laparoscopy revealed the presence of adhesions at the site of origin of the MD, leading to distal ileal luminal narrowing. An urgent trans-umbilical laparoscopic removal of the MD and small bowel resection was performed.

This case series describes the varied presentations of MD on CECT. One of the cases was incidentally diagnosed and the diverticulum was asymptomatic. Two patients presented with an intestinal obstruction, one with diverticulitis and one with perforation.

## Discussion

In 1809, Johann Friedrich Meckel, the Younger, described the most common congenital anomaly of the gastrointestinal tract and its anatomy, embryology and clinical features.^3^ The omphalomesenteric duct (OMD) connects the yolk sac with the developing midgut in foetal life. During the 10th to 12th week of the intrauterine period, this duct gradually involutes when the midgut returns to its normal position.^4^ Depending on the degree of failure of involution of the OMD, different types of anomalies develop. Omphalomesenteric duct anomalies include umbilical sinus, Meckel’s diverticulum, omphalomesenteric cyst, a fibrous cord connecting the ileum to the umbilicus and ileo-umbilical fistula. Umbilical-ileal fistula is the least common anomaly presenting in the newborn period, resulting from a completely patent OMD.^4^ Meckel’s diverticulum is the most common, accounting for 98% of OMD anomalies, which occurs as a result of persistence of the duct at its ileal end.^4^

People with MD remain mostly asymptomatic during their lifetime. Asymptomatic MD is often incidentally diagnosed on imaging studies or intraoperatively during surgeries. Only about 16% – 20% MD becomes symptomatic secondary to one or other complications.^5^ Complications are also more common in the paediatric age group before 10 years.^5^ Different studies have shown that the lifetime risk of complications varies between 4% – 6%.^6^ Meckel’s diverticulum is generally composed of small intestinal mucosa; however, they frequently develop heterotopic tissue, especially gastric and pancreatic mucosa, which leads to peptic ulceration and other complications. Rarely duodenal, colonic, jejunal and biliary ectopic tissue may be present in them.

The most common complication of MD is lower gastrointestinal tract haemorrhage presenting as painless rectal bleeding, especially in paediatric patients.^7^ Sometimes this can clinically present as prolonged anaemia with a positive occult stool blood test or episodic per rectal bleeding. Haemorrhage occurs secondary to peptic ulceration of the MD and adjoining ileal mucosa from the acidic or alkaline secretions of gastric or pancreatic ectopic tissue in the MD.^7^

Intestinal obstruction is the second most common complication, particularly in adults. Clinically, it manifests as bilious vomiting, constipation, abdominal pain and distension. The mechanism for the development of intestinal obstruction in MD may be intussusception, volvulus or internal herniation secondary to a fibrous band connecting the MD with the umbilicus, diverticular inversion, meso-diverticular band, adhesions or diverticulitis leading to stricture. The inclusion of MD in an inguinal hernia is known as Littre hernia. Foreign body or stone impaction and a neoplastic lesion can also cause obstruction.^8^

Acute Meckel’s diverticulitis is the third most common complication presenting as acute abdominal pain, fever and vomiting. Its clinical symptoms closely mimic acute appendicitis and present a diagnostic challenge. It is often misdiagnosed preoperatively and only identified during surgery. It develops because of narrowing of the MD at its origin by a faecolith, foreign body, calculus, neoplasm, secretions or inflammation and stricture formation secondary to peptic ulceration.^8^ Enteroliths are seen in up to 10% of cases of MD, especially in the adult age group and clinically present as chronic dull aching paraumbilical pain.^8^ Perforation is a less common but severe and life-threatening complication of MD. It occurs secondary to peptic ulceration, diverticulitis and gangrene. Patients present with severe abdominal pain, tenderness, rigidity and poor general condition. Thus, the clinical suspicion of MD should always be kept in mind in a young patient presenting with clinical features related to its complication.

Imaging plays a crucial role in the diagnosis of a MD. Plain X-ray abdomen and fluoroscopic studies have a limited role in identifying MD except for identifying complications such as perforation, bowel obstruction, intussusception and volvulus. On barium studies, the characteristic findings described are a triradiate bowel fold of the diverticulum in the presence of collapsed bowel, mucosal triangular plateau appearance in the presence of distended bowel and as bulbous or triangular area in the setting of intussusception or inverted diverticulum.^9^ However, these findings are difficult to interpret and not confirmatory. Ultrasound is a part of the initial radiological evaluation of MD complications. On ultrasound, an inflamed MD appears as a non-compressible, irregular, thick-walled, cystic mass with increased vascularity and echogenicity of the surrounding fat.^9^ The typical gut signature appearance of its wall is seen on ultrasound. This appearance can be easily confused with acute appendicitis; therefore, identification of a normal appendix on ultrasound is essential. An inverted MD with intussusception produces a target-like appearance on ultrasound; however, these findings lack specificity for MD.

The CT scan is the modality of choice for identifying a MD presenting with complications, especially in adults. When combined with enteroclysis or enterography, CT has added benefits in the delineation of MD.^10^ Meckel’s diverticulum is usually identified as a blind-ending thick-walled tubular structure arising from the antimesenteric border of the ileum, usually within 100 cm of the ileocaecal valve on CT. It is supplied by a persistent vitellointestinal artery arising from the superior mesenteric artery.^11^ Connection of this blind-ending structure with the umbilicus through a thin linear track also favours the diagnosis of MD. Identification of a normal appendix on CT makes the diagnosis of MD more confident.

Meckel’s diverticulitis presents as an irregular thickened enhancing wall of a dilated MD with surrounding mesenteric fat inflammation at CT. Surrounding abscess, fluid collection and enlarged lymph nodes may be seen. Enteroliths can also be seen in the MD, causing diverticulitis and obstruction. Chronic Meckel’s diverticulitis can produce a mucocele-like appearance on CT. Enteric duplication cyst can have a similar appearance on CT; however, there is no bowel communication and smooth walls are seen with duplication cysts. The typical antimesenteric location of MD differentiates it from enteric diverticulitis. In the case of an inverted Meckel, a central core of fat attenuation surrounded by a rim of soft-tissue attenuation differentiate it from lipoma. Obstruction occurring secondary to MD requires a high degree of suspicion and careful evaluation of the site of transition.

The CT angiogram has moderate sensitivity in identifying active extravasation of contrast from an actively bleeding MD.^8^ A Tc-99m pertechnetate scan is the modality of choice and most specific diagnostic test for evaluating haemorrhage if there is a suspected MD, especially in the paediatric population.^7^ The tracer accumulates in the ectopic gastric mucosa of the MD, which is responsible for the bleeding. The Tc-99m–labelled sulfur colloid or red blood cell scintigraphy is sensitive for the localisation of the site of gastrointestinal haemorrhage occurring at a very low rate; however, these methods are not specific for MD. Conventional angiography is very useful in adults with intermittent gastrointestinal bleeding.^11^ It can localise the site of active bleeding, confirm the presence of MD by demonstrating its blood supply from the vitellointestinal artery arising from a distal ileal branch of the superior mesenteric artery and allow access for therapeutic embolisation.

Laparoscopic resection of the diverticulum is the treatment of choice for symptomatic MD.^12^ In some complicated conditions, laparotomy and resection may be required. Treatment of asymptomatic and incidentally diagnosed MD is controversial. Whilst some authors advocate prophylactic resection because of the risk of complication later in life, others disagree.^13^ A published study reported a risk score for prophylactic resection of asymptomatic MD, which included risk factors of developing complications such as male gender, age younger than 40, diverticula over 2 cm in diameter, suspected heterotopic tissue, associated meso-diverticular band and wide diverticula with thin walls.^14^

## Conclusion

Although MD is the most common gastrointestinal tract anomaly, its preoperative diagnosis is rarely made. A myriad of complications can develop in MD, making them symptomatic. Clinical symptoms of complicated MD are non-specific and mimic other common pathologies of the abdomen causing acute or subacute pain. Therefore, a high clinical index of suspicion of MD should always be maintained in patients in whom the cause of acute abdomen, obstruction, lower gastro-intestinal (GI) haemorrhage and perforation is not found. Computed tomography combined with enteroclysis or enterography is the investigation of choice for the preoperative diagnosis of complicated MD. This case series highlights the importance of CT in demonstrating MD and its complications. Knowledge of the clinical features, characteristic imaging appearance of MD and its different complications will aid in its accurate and early preoperative diagnosis.
